# Supplementary material for: Beat perception in polyrhythms: Time is structured in binary units
Source: PLoS One. 2021 Aug 20;16(8):e0252174. doi: 10.1371/journal.pone.0252174 (PMC8378699; doi:10.1371/journal.pone.0252174)
Supplement: Attachment — Submitted filename: polyrhythm_response_to_reviewers_revision_I.docx [file pone.0252174.s002.docx]

Response to Reviewers, Revision I

**Beat Perception in Polyrhythms: Time is Structured in Binary Units**

Cecilie Møller*, Jan Stupacher*, Alexandre Celma-Miralles*, and Peter Vuust

# Reviewer #1:

In this manuscript, the authors deploy a massively crowdsourced tapping study to polyrhythmic auditory stimuli, and use the results to argue that subdivision structure is a key determining factor in where human listeners perceive a beat and tap along. Specifically, they claim that listeners are strongly biased toward identifying beat structures such that events between beats subdivide the beat into two rather than three or more even parts.

The argument is clear, cohesive, and well supported by the data. It adds an important and often overlooked dimension to the discussion of beat perception. The hypothesis and the insightful discussion around it are well articulated and seem to be profoundly informed by strong musical intuition. Overall, I believe it is a valuable contribution to the field that will help shape the discussion moving forward.

The formidable challenge these authors faced in communicating these results is navigating the many pieces of information necessary to fully characterize each tapping pattern — tempo, subdivision level, subdivision duration, whether it the tap is the faster or slower click track or a doubled or halved version of one of them. In Figure 1, they do an excellent job defining useful terms, which they use consistently throughout the paper. However, as they discuss their results in the text and display them in figures, it sometimes becomes difficult for the reader to map the specific tap pattern categories onto the take-away messages about subdivision. I would like to see the authors work to communicate their key message more clearly through language and image. Here are some example suggestions:

* At some points in the text, they refer to the “slow pulse train” and then give the subdivision level (binary/ternary) in parentheses — I would strongly encourage the authors to use such a convention systematically throughout, and to go even further when appropriate by specifying, e.g., (ternary, 480ms tap period, 120ms subdivisions). I recognize that this could lead to clutter, but I think it’s at least worth taking another step in this direction in the Results section.

REPLY: This is a very useful recommendation. We have implemented this format at the relevant places in the revised manuscript.

* Looking over Figure 4, it is not immediately clear which tap patterns are associated with binary and which with ternary subdivisions. Perhaps a color coding convention would help, or at least careful labeling, to highlight the tendency toward binary subdivision, which is not readily observable in this figure without much careful interpretation.

REPLY: Thanks, this is also a great suggestion. In the revised manuscript we color-coded Figure 4 corresponding to binary vs ternary subdivision of the beat. The colors for binary and ternary subdivision grouping are the same as in Figure 3. In the note of Figure 3, we now refer to the color coded categories in Figure 4.

Figure 4 Note (relevant changes in **bold)**:

*“Note.* Bars represent the number of participants tapping in time with each of the eight different metrical categories ranging from the fastest category, *all subdivisions* (top) to the slowest category, *cycle* (bottom). Responses to the 2:3 and the 3:4 polyrhythms are shown at different tempi ranging from slow (left) to fast (right). Individual x-axes show the tapping tempo of responses included in that metrical category. For instance, a participant listening to the slowest 2:3 polyrhythm may choose to tap in time with either *all subdivisions* at 120 BPM, the *slow pulse train - double tempo* at 80 BPM, the *fast pulse train* at 60 BPM, the *slow pulse train* at 40 BPM, the *fast pulse train - half tempo* at 30 BPM, or the *cycle* level at 20 BPM. **The grey-shaded areas mark stimuli with tempi at which the subdivision tempo exceeds 600 BPM / 100 ms, i.e., the cognitive limit of subjective grouping of sounds (London 2002; Repp, 2003).** Double asterisks (**) mark significant Cochran’s Q tests comparing responses over different tempi within each metrical category. Asterisks (*) mark significant McNemar’s tests for pairwise comparisons between neighboring tempi within each metrical category. All tests are Bonferroni-corrected for multiple comparisons.”

Figure 3 Note:

*“Note.* The different lines in the plot represent responses with binary (dark purple) or ternary (light green) subdivision grouping. At slow and moderate tempi, most participants tapped in time with one of the response categories that admitted a binary subdivision regardless of tempo. At faster tempi, participants switched to tapping in time with the cycle. Only a few participants’ tapping behavior corresponded to ternary subdivision grouping. Binary subdivision in 2:3 include *fast pulse train*, *fast pulse train - half tempo*. Binary subdivision in 3:4 include *slow pulse train*, *slow pulse train - half tempo*, *slow pulse train - double tempo*, and *fast pulse train - half tempo*. Ternary subdivision in 2:3 include *slow pulse train* and *slow pulse train- double tempo*. Ternary subdivision in 3:4 include *fast pulse train* and *fast pulse train - double tempo***. For an overview of binary and ternary metrical categories, see also the color-coded tapping responses in Figure 4.** Note that subdivision grouping is indiscernible at the *all subdivisions* (not shown) and *cycle* levels.”

* The language in Figure 5 is confusing — “tapping expected to be related to the slow pulse train” is a strange way of saying “slow pulse train admits binary subdivision"

REPLY: We chose this formulation because the pulse trains in the different polyrhythm ratios are not only in a binary:ternary relation but also in ternary:irregular and binary:irregular relations. However, we agree that the formulation might be confusing and changed it to “... pulse train admits simpler subdivision”. Thanks for this suggestion!

Additional concerns:

The number of trials excluded is pretty substantial. I would like to see some discussion of what was going on in those cases, or at least assurance that the exclusion process is not skewing the data.

We agree to include more details about the pre-processing and analyses of tapping responses. The figure below describes the percentage of responses that our pipeline labeled as *categorized* (i.e., in one of the following metrical categories: cycle, all subdivisions, fast or slow pulse train, and their half and double tempi), *inconsistent* (with ITIs beyond ±15% and/or standard deviation beyond 66% of the IOI of the assigned meter), *uncategorized* (non-significant Rayleigh nor Rao’s Spacing tests), *excluded* (with less than 7 taps after removing double taps) and *missing* (without any recorded tapping responses) for each experiment (Tempo, Ratio, Pitch). We included the figure in the Supplementary Material, Figure S1. We also added these details in the Methods section of the MS, i.e., in the first and last paragraph of Finger Tapping Analyses:

“We obtained a high proportion of tapping responses. Only 1.3% of the tapping trials were missing in Tempo, 2.0% in Ratio, and 1.0% in Pitch. To remove involuntary double taps and device artefacts, we calculated all the time intervals between consecutive taps (Inter Tapping Intervals, ITIs) and removed the second tap of each ITI shorter than 150 ms (see figure S1A in Supplementary Material). We additionally removed the first 2 taps of each trial. If less than five taps remained, we removed the trial from the analysis. The resulting percentages of **excluded trials in this step were 3.7% in Tempo, 4.6% in Ratio, and 3.0% in Pitch.”**

[...]

“Using circular statistics (Berens, 2009) we computed the mean resultant vector of the tapping responses to each stimulus for every metrical level. The tapping consistency is reflected by the length of the mean vector ranging from 0 to 1. To determine the metrical level tapped by the participant in each trial, we took the longest vector length among all the metrical levels and checked whether the taps were uniformly distributed using the Rao’s Spacing test and the Rayleigh test. Only when both tests were significant (p ≤ .05), the tapping responses were assigned to a metrical category. **This procedure filtered out non-regular tapping responses, including irregular grouping of subdivisions and synchronization with the rhythm itself. The percentages of trials not assigned to any metrical category (i.e. non-significant at least in one of the two circular tests) were 12.3% in Tempo, 24.7% in Ratio, and 5.1% in Pitch. The larger proportion of uncategorized responses in the Ratio Experiment reflects the increased complexity of the stimuli presented here.** Finally, we confirmed the selection of the categorized metrical level by only accepting those metrical tapping responses (i) whose mean of the ITIs fell in the range of ±15 % of the inter-onset interval (IOI), i.e., the tempo in milliseconds, of the categorized meter and (ii) whose standard deviation of the ITIs was smaller than 66% of the IOI. These means and standard deviations were obtained after removing ITIs that fell beyond two standard deviations from the mean ITI of each trial. **The percentages of trials rejected in this step were 16.6% in Tempo, 14.7% in Ratio, and 14.7% in Pitch.** The combination of linear and circular analyses resulted in **the final** inclusion of the following percentages of trials with consistent tapping at one of the metrical levels: 66.2% in Tempo, 54.1% in Ratio, and 76.2% in Pitch. **See Figure S3 in Supplementary Material for visualization of trials excluded in each of the data cleaning steps.”**

**Figure S3.** Percentage of tapping responses for each experiment (Tempo, Ratio, Pitch) that our pipeline labeled as *categorized* (i.e., one of the following metrical categories: cycle, all subdivisions, fast or slow pulse train, and their half and double tempi), *inconsistent* (with ITIs beyond ±15% and/or standard deviation beyond 66% of the IOI of the assigned metrical tempo), *uncategorized* (non-significant Rayleigh nor Rao’s Spacing tests), *excluded* (with less than 7 taps after removing double taps), or *missing* (without any recorded tapping responses).

I find it a little confusing that the authors lump all even subdivision patterns in with binary subdivision. I would appreciate a little more clarity in the discussions of tapping patterns that have 4, 6, etc. subdivisions —they call these “binary subdivision groupings,” but the 6 group could equally be called ternary. Perhaps language like “admit a binary subdivision” might be more precise?

REPLY: We consider grouping of four subdivisions binary but we do not consider six subdivisions binary or ternary for the reason stated by the reviewer. Perhaps the concern was raised because of the 3:4 polyhrythm’s *fast pulse train - half tempo* category. Indeed, here it appears that six subdivisions are grouped and considered binary. However, as explained in the Tempo Experiment, Results and Discussion, responses in this category fell at rates that exceed the cognitive limit of subjective grouping of sounds, i.e., rates where the pulse train itself is perceived as the subdivision level (4 subdivisions = binary). We have made this clearer in the following addition to this section and highlighted the relevant area in grey in Figure 4 (see also Figure 4 in our reply to a previous comment):

“Participants discarded the most common tapping preference, i.e., *fast pulse train* in 2:3 (binary, 304 BPM / 198 ms tap period, 608 BPM / 99 ms subdivisions) and *slow pulse train* in 3:4 (binary, 160 BPM / 375 ms tap period, 640 BPM / 94 ms subdivisions) around this point. **In the 3:4 polyrhythm, *fast pulse train - half tempo* became the preferred response, indicating that the fast tempo caused participants to group the elements of the pulse train rather than their subdivisions, i.e., tap every other beat of the 4-pulse train**. Furthermore, a significant shift was observed from *fast pulse train - half tempo* (binary, 253 BPM / 237 ms tap period, 506 BPM / 119 ms subdivisions) to *cycle* (indistinguishable, 126 BPM / 476 ms tap period) at the fastest tempo of the 3:4, where even the pulse train (at 506 BPM / 119 ms) approached the cognitive limit of subjective grouping of short sounds.”

We have been unable to find a place in the manuscript where we referred to six subdivisions as binary. We stick with descriptions such as “5:6 (binary/ternary:irregular)”. See also our response to the Figure 5 comment above concerning our use of “simpler” in cases where binary subdivision is not inherent in the metrical structure of the polyrhythm.

To clarify further, we added the following sentence to the Methods section of the Ratio experiment:

“Accordingly, with respect to subdivision grouping, the 2:3 polyrhythm can be denoted ternary:binary, while for instance the 2:5 polyrhythm can be denoted irregular:binary. **Because six subdivisions may be grouped in either two or three, the 5:6 polyrhythm should be denoted binary/ternary:irregular.”**

Finally, the data were not actually available to me — it seems that they were password protected, and I did not have a “review key” with which to view them.

REPLY: This was sorted out already. FYI, once a researchbox.org is made public, only limited info can be added which was why we waited for confirmation from our IRB that we could share the data. Before making it public, reviewers can access the box with a password included in the URL. Because we uploaded a preprint with the submission, this reviewer link was only sent as a comment to the editorial office. We have now made the box public and the link provided in the manuscript gives access to the content: [https://researchbox.org/278](https://researchbox.org/278&PEER_REVIEW_passcode=XXGVLC)

# Reviewer #2:

The experiments per se were well performed and analyzed. However, see my main concern below, some concepts are confusing, and the rationale of the exact question to be addressed, as well as how the conclusion is supported, are unclear.

Main concern:

For a common basis of concepts and terminology to discuss. A meter can be subdivided into beats (e.g., a 2/4 meter or a 3/4 meter), and a beat can also be subdivided into lower levels (e.g., into 2, 3 subdivisions). The preference of binary over ternary subdivision, on both the meter or the beat level, has been studied. Given a singer and a pianist who play together, they follow the same meter, i.e., the same subdivision on the meter level; may or may not follow the same subdivision on the beat level. This is a common situation, in which they had a consistent metrical structure (here, yes, subdivision on the beat level can be different), which had the same sense of beat that allows them to play synchronously.

In the manuscript, terms such as “subdivision”, “metrical structure”, “metrical level”, are ambiguously used, in terms of meter of beat. For example, Fig. 1A, what “circle” refers to?

REPLY: The terms mentioned are defined in the 4th paragraph of the Introduction of the original manuscript and used consistently throughout the manuscript:

*“The regular pulse we emphasize when synchronizing with music represents only one level in a more complex metrical structure. Figure 1 explains the concept of a metrical structure and illustrates its perceptual and behavioral consequences. Figure 1A illustrates how the subdivisions mark the points of the metrical grid, which is established on the basis of the smallest interval between perceptible events of the stimulus. The beat level is the level with which we usually synchronize our body movements. The cycle level marks the onset of the whole repeating pattern. Although the beat level is often the most perceptually salient level, we exhibit high flexibility with regards to synchronizing with any level in the metrical structure (Large et al., 2002). What is considered moving “in time with music” can relate to any level of the metrical structure. [...]”*

If meter (I guess), then why, or in what kind of situation, would one play in a 2/4 meter and another plays in a 3/4 meter? In other words, why the referred “2:3” polyrhythm is a question to address? If beat, yes, people often do it, also the manuscript describes “are often used to create tension and increase expressiveness in musical performances”. But again, as above, it is common for same subdivision on the meter level and same/or different subdivision on the beat level. What is the exact question?

REPLY: Perhaps our reply to the comment above helped to solve the reviewer’s concern? See also the definition and example of a polyrhythm in the original manuscript (Introduction, 5th paragraph):

*“In the special case of polyrhythms, two or more metrical structures co-exist and as such, polyrhythms are often used to create tension and increase expressiveness in musical performances. A polyrhythm is created by presenting at least two pulse trains containing coprime numbers of beats within the same periodic cycle, e.g., in ratios of 2:3, 3:4, or 3:5. A listener may perceive one or the other pulse train as representing the underlying beat and extract the corresponding metrical structure. The example depicted in Figure 1 is a 2:3 polyrhythm. What distinguishes the two manifestations of metrical structure is the beat level, which is defined by the grouping of elements at the subdivision level. On the left of Figure 1A, the ternary subdivision grouping results in two perceived beats per cycle. On the right, the binary subdivision grouping results in three perceived beats per cycle. The resulting metrical structures are organized differently and give rise to distinct and mutually exclusive perceptual experiences depending on how the elements at the subdivision level are grouped (Figure 1B). Two of these perceptual experiences are illustrated with speech examples in Figure 1C.”*

It seems that subdivision on beat level and subdivision on meter level are confused. The corresponding descriptions and statements are ambiguous and confusing, e.g., “a yet neglected compelling influence of beat subdivisions on beat perception”, “Our findings highlight the importance of metrical structures, which need to be taken into account in order to understand beat perception—an insight that has been overlooked in previous polyrhythm studies.The authors please explain the rationale of the study, what exactly is the question. Perhaps, previous studies did not neglect or overlook the referred question.

REPLY: The study assessed factors influencing beat perception in polyrhythms as indicated by tapping responses. Contrary to previous studies, we focused on grouping of subdivisions. Hence, we could show a preference for simpler subdivision groupings that was not evident in previous studies that focused on the number of elements in the constituent pulse train of different polyrhythms. This is explained in the Introduction and reiterated in the General Discussion of the original manuscript.

We explained the rationale of the studies in the Introduction 9th paragraph (original manuscript):

*“To provide a comprehensive account of beat perception that takes into account the most basic level of the metrical hierarchy, the purpose of this online finger tapping study was to assess how subdivision grouping biases listeners to adopt one rather than another possible metrical structure inherent in a given polyrhythm consisting of two pulse trains. Owing to their ambiguous nature, polyrhythms are ideal stimuli for assessing rhythmic interpretations in tapping studies. Such studies rest on the assumption that subjects synchronize their taps with the perceived beat (Beauvillain, 1983; McKinney & Moelants, 2006). The paradigm in the present study allowed for categorization of tapping responses into all possible metrical levels, including half and double tempo in relation to the constituent pulse trains. Overall, we hypothesized that participants would prefer to tap to a beat with binary rather than ternary or irregular subdivision grouping, and ternary rather than irregular subdivision grouping. This hypothesis was based on the general propensity for binary grouping of isochronous auditory stimuli and their subdivisions (e.g., Bolton, 1894; Brochard et al., 2003; Repp, 2008).”*

And in the General Discussion, 3rd paragraph, we related our findings to seminal work on beat perception in polyrhythms which did not take beat subdivisions into account:

“*However, we do not agree with Handel’s notion that “There are no basic units of rhythm, and no theories based on grouping principles or tree representations seem able to encompass rhythmic diversity” (Handel, 1984, p. 481). Our Tempo Experiment showed that binary grouping of subdivisions was preferred regardless of whether the beat corresponded to the fast or the slow pulse train (Figures 3 and 4). Thus, a principle of simpler grouping pertains to the subdivisions, not the beats, making the subdivision the basic unit of rhythm.”*

We acknowledge that getting an exhaustive overview of the many possible regular beats that can be extracted from polyrhythms may be challenging for readers outside the field of music. To further facilitate comprehension and identification of the many regularities inherent at the different levels of the competing metrical structures, we have added a figure, Figure S1 in Supplementary Material, showing the options of interpretations that an individual is faced with when listening to a 2:3 polyrhythm**.**

**Figure S1.** When listening to music we spontaneously extract a regular pulse. Polyrhythms include at least two regular pulses, each representing mutually exclusive metrical structures. In this example of a 2:3 polyrhythm, the two pulse trains—2 and 3, depicted in the stimulus boxes above—support two different perceptual experiences corresponding to two kinds of subdivision grouping: ternary (groups of three subdivisions as displayed on the left side) and binary (groups of two subdivisions as displayed on the right side). Finger tapping in time with a polyrhythm is an observable outcome of our interpretation, which depends on whether we perceive ternary (left) or binary (right) subdivisions. Depending on how fast the polyrhythm is presented, we can tap in time with different metrical levels, e.g. all subdivisions with slow stimuli, pulse train with moderately fast stimuli, and cycle with fast stimuli. Note that in this example, when tapping fast pulse train - half tempo with binary subdivision grouping (right) it is necessary to concatenate two cycles.

# Reviewer #3:

The authors explore beat perception in polyrhythms through three online finger tapping experiments and make an interesting claim that beat, rather than being primarily constrained by tempo (as is widely thought in the field), is primarily constrained by subdivisions, with a strong preference for binary groupings. This claim/perspective is very intriguing and important for the field. The paper is also very well written and presented. In my opinion, the work definitely deserves to be published, but I have a few concerns.

My biggest concern is the highly "Western" makeup of participants, because perhaps the conclusion that "we prefer to structure time in binary units" could be a result of the tendency of Western popular music to be in binary meters. To me, the data presented are not sufficient to conclude that we (=all humans) biologically have this binary bias. However, I appreciate this perspective proposed by the authors, so perhaps a deeper discussion of the limitations of the demographics of the sample and/or an additional analysis of the countries/languages/background of the minority of participants who preferred ternary groupings, would add some necessary nuance to the central claim of the paper. Along these lines, the cross-cultural iterated reproduction study of Jacoby & McDermott 2017 is not mentioned at all - how consistent is cross-cultural work with the idea of a universal preference for binary subdivisions?

REPLY: This is a relevant point! As stated in the original manuscript, we will address questions related to personal and cultural backgrounds of participants in a second study that will include all the analysis mentioned in our preregistration:

*“In future studies, we will expand the current findings in a number of exploratory analyses mentioned in the preregistration for the present study (https://aspredicted.org/yi5si.pdf). We will examine the influences of musical training, individual spontaneous motor tempo, native languages, and cultural background on subdivision grouping preferences in rhythm processing.“*

In this manuscript we chose to stay true to the main analyses described in the preregistration. That said, we realize that the question of musical culture is likely the first one asked by readers of this work, so we have revised the manuscript to accommodate these concerns.

We have changed the formulation of the sentence in cases where “we” could be understood as “all humans”, i.e.,

End of Pitch, Results and Discussion:

“Taken together, **our findings show that participants** preferred to synchronize movements with polyrhythm pulse trains that consist of lower pitched sounds and simpler groupings of subdivisions.”

End of General Discussion:

“Studying the hierarchical organization of temporal patterns is an interdisciplinary effort. From the perspective of music perception and cognition, our findings reveal the importance of lower hierarchical levels, such as subdivisions, to grouping processes at higher levels, such as beat and meter. Our study adds to evidence suggesting that in everyday behavior, **time is structured in binary units.”**

In addition, we have added the following section to the general Discussion, which also incorporates our additions following your comments below on the findings of Savage et al 2015 regarding binary vs. ternary meters in music across cultures and possible neurophysiological accounts for the preference for binary subdivision.

“A growing body of research suggests that experience within a musical culture shapes perception and consistency of production of musical rhythms prevalent in that culture (Jacoby & McDermott, 2017; Polak et al., 2018). A pivotal question concerns the universality of the proposed preference for binary subdivision grouping, considering the large cross-cultural variation in music traditions. Our study sample consisted mainly of Western participants (Western Europe, North America). Simple binary rhythms are dominating the Western musical culture whereas more multifaceted rhythmic patterns are prevalent in non-Western musical cultures, e.g., in the Balkans, Africa and Latin America (for an overview see e.g., Haumann et al., 2018). Not only do West African drummers exhibit considerable rhythmic flexibility in terms of switching between metrical levels of the typical 12-unit rhythmic patterns, they are also more likely to perceive four regular beats, i.e., with ternary subdivision of the beat (Locke, 1982). In contrast, the participants in the present study perceived three regular beats with four subdivisions in the comparable 12-unit 3:4 polyrhythm, and avoided synchronizing with the fast pulse train that admitted a ternary subdivision. The lack of cultural variation in our participant sample is clearly a limitation. However, a recent cross-cultural study shows that perceptual priors representing small-integer ratio rhythm categories are universal (Jacoby et al., 2021). Given the universality of simple subdivision grouping, a remaining key question is whether the contrasting beat perception in the two populations reflects differences in metrical flexibility. In other words, the simplest grouping patterns are present even in musical cultures with rich, multifaceted rhythmic patterns, and members of these cultures are likely more rhythmically adept. Importantly, musical training is also associated with greater metrical flexibility as indicated by studies showing that trained musicians demonstrate more complete hierarchical representations of the rhythmic structure of music (Drake et al., 2000; Geiser et al., 2010; Vuust et al., 2005). Perhaps the lack of metrical flexibility constrains the tapping responses of Western listeners to correspond to the simplest possible grouping of subdivisions, whereas e.g., West African listeners can more easily switch between metrical levels (Locke, 1982).”

The other aspect of this paper that needs improvement is the terminology around the different tapping patterns, which becomes increasingly confusing. For example, in Fig 3: "Tapping responses related to groupings of binary and ternary subdivisions are concatenated separately": what does this and the text that follows mean? You also say "binary grouping in 2:3 (fast pulse train including half tempo)", then also "binary grouping in 3:4 (slow pulse train, including half tempo and double tempo, and fast pulse train - half tempo) it is very challenging to follow this terminology. Fig. 1 does an excellent job of illustrating the concepts of subdivision, beat, cycle, and stimulus - perhaps fig 1 (or some version of it) could be expanded to include visually showing the tapping pattern that each of the tapping patterns in fig 4 corresponds to.

REPLY:

REPLY: Thank you for pointing to these important aspects of communicating our results. Reviewer 1 had the similar concerns so parts of this reply are copy-pasted from above. We updated the Note of Figure 3 and color-coded Figure 4 in the revised manuscript (same colors as in Figure 3).

.

Figure 4 Note (relevant changes in **bold)**:

*“Note.* Bars represent the number of participants tapping in time with each of the eight different metrical categories ranging from the fastest category, *all subdivisions* (top) to the slowest category, *cycle* (bottom). Responses to the 2:3 and the 3:4 polyrhythms are shown at different tempi ranging from slow (left) to fast (right). Individual x-axes show the tapping tempo of responses included in that metrical category. For instance, a participant listening to the slowest 2:3 polyrhythm may choose to tap in time with either *all subdivisions* at 120 BPM, the *slow pulse train - double tempo* at 80 BPM, the *fast pulse train* at 60 BPM, the *slow pulse train* at 40 BPM, the *fast pulse train - half tempo* at 30 BPM, or the *cycle* level at 20 BPM. **The grey-shaded areas mark stimuli with tempi at which the subdivision tempo exceeds 600 BPM / 100 ms, i.e., the cognitive limit of subjective grouping of sounds (London 2002; Repp, 2003).** Double asterisks (**) mark significant Cochran’s Q tests comparing responses over different tempi within each metrical category. Asterisks (*) mark significant McNemar’s tests for pairwise comparisons between neighboring tempi within each metrical category. All tests are Bonferroni-corrected for multiple comparisons.”

Figure 3 Note:

*“Note.* The different lines in the plot represent responses with binary (dark purple) or ternary (light green) subdivision grouping. At slow and moderate tempi, most participants tapped in time with one of the response categories that admitted a binary subdivision regardless of tempo. At faster tempi, participants switched to tapping in time with the cycle. Only a few participants’ tapping behavior corresponded to ternary subdivision grouping. Binary subdivision in 2:3 include *fast pulse train*, *fast pulse train - half tempo*. Binary subdivision in 3:4 include *slow pulse train*, *slow pulse train - half tempo*, *slow pulse train - double tempo*, and *fast pulse train - half tempo*. Ternary subdivision in 2:3 include *slow pulse train* and *slow pulse train- double tempo*. Ternary subdivision in 3:4 include *fast pulse train* and *fast pulse train - double tempo***. For an overview of binary and ternary metrical categories, see also the color-coded tapping responses in Figure 4.** Note that subdivision grouping is indiscernible at the *all subdivisions* (not shown) and *cycle* levels.”

Thank you for suggesting a new figure. Figure S1, Supplementary Material now shows examples of the tapping behavior for the different metrical categories in the 2:3 polyrhythm.

**Figure S1.** When listening to music we spontaneously extract a regular pulse. Polyrhythms include at least two regular pulses, each representing mutually exclusive metrical structures. In this example of a 2:3 polyrhythm, the two pulse trains—2 and 3, depicted in the stimulus boxes above—support two different perceptual experiences corresponding to two kinds of subdivision grouping: ternary (groups of three subdivisions as displayed on the left side) and binary (groups of two subdivisions as displayed on the right side). Finger tapping in time with a polyrhythm is an observable outcome of our interpretation, which depends on whether we perceive ternary (left) or binary (right) subdivisions. Depending on how fast the polyrhythm is presented, we can tap in time with different metrical levels, e.g. all subdivisions with slow stimuli, pulse train with moderately fast stimuli, and cycle with fast stimuli. Note that in this example, when tapping fast pulse train - half tempo with binary subdivision grouping (right) it is necessary to concatenate two cycles.

Other questions:

- how were taps collected? were they laptop key presses? how reliable were tap times? how much delay/jitter from the operating system or browser?

REPLY: Thanks for noticing that this important info was not included. Participants tapped on their touchscreen, touch pad or mouse. This information has been added to the Procedure section.

“After a spontaneous motor tempo assessment, which familiarized participants with tapping on their device **(touchscreen, touch pad, or mouse)**, the experimental tapping task was explained.”

Because every operating system, browser and tapping device has different delays, which are assumed consistent within and between trials, we used ITIs and circular statistics for data cleaning and analysis. These analyses do not look at the stimulus-tapping phase and are not affected by delays. We added the following sentence (in bold) to the Finger Tapping Analyses section:

“This method allowed us to obtain the consistency of the taps at each metrical level, even if the device or the participant missed some taps. **Because every operating system, browser and tapping device has different delays, we did not analyze stimulus-tapping phase but used ITIs and circular statistics**.”

- the inclusion criteria for trials seems quite strict, especially if only 54.1% of trials were finally included in the Ratio experiment. Could there be higher motor error while tapping to irregular / ternary groupings, and could that cause trials to be excluded, effectively biasing data in favor of binary?

This is a good point. Similar details were asked for by Reviewer 1. We added a new Figure (Figure S3, see below) to Supplementary Material. The figure provides detailed percentages of included/excluded trials at every step of the preprocessing and categorization of the tapping trials. We rephrased two paragraphs in the Methods section to clarify the percentages of inclusion and categorization of tapping responses (see also response to Reviewer 1’s *Additional concerns*).

**Figure S3.** Percentage of tapping responses for each experiment (Tempo, Ratio, Pitch) that our pipeline labeled as *categorized* (i.e., one of the following metrical categories: cycle, all subdivisions, fast or slow pulse train, and their half and double tempi), *inconsistent* (with ITIs beyond ±15% and/or standard deviation beyond 66% of the IOI of the assigned metrical tempo), *uncategorized* (non-significant Rayleigh nor Rao’s Spacing tests), *excluded* (with less than 7 taps after removing double taps), or *missing* (without any recorded tapping responses).

Regarding the Ratio experiment, it is possible that tapping to the beat that grouped irregular/ternary subdivisions could have led to higher motor errors, and therefore more exclusion of trials. However, a large part of our trial exclusions are related to device artefacts and to participants imitating the rhythm itself (as is often seen in online tapping studies), and even if all remaining excluded trials represent participants trying to tap to a beat with ternary subdivisions, it is unlikely that such exclusions are driving the results. Since these trials were not categorized, we cannot identify them objectively to compare the variance between ternary/irregular-grouping and binary-grouping responses.

- pitch task: was the 2-octave choice deliberate? why? in this case, low and high tones are similar perceptually, in the sense that they would fuse perceptually if played at the same time. would a more dissonant/contrasting higher pitch (e.g. tritone) have been more effective at attracting synchronization?

REPLY: Yes, we carefully thought about which interval to use to assess the effect of low frequency (pitch) on beat perception. We agree that dissonance would have maximized segregation of the two pulse trains and this is exactly the reason why we decided to not use a dissonant interval. We also agree that two octaves result in high perceptual similarity. And again, this is exactly the reason why we chose two octaves: the effect is driven by the bass frequency - even with perceptually similar tones.

- the discussion of Handel and Oshinsky (1981) is incomplete... why were their results were different? different demographic of participants? differences in stimuli?

REPLY: We gave this a lot of thought even before collecting our own data as we struggled with interpreting the distribution of responses reported in Handel and Oshinsky (1981). Frankly, we haven’t been able to find a straightforward explanation since then, but we have added the following “best suggestion” to the Tempo Experiment, Results and Discussion section in the revised manuscript.

“The reasons for the discrepancies between our findings and the ones of Handel and Oshinsky are not immediately obvious. One reason could be that the categorization of responses differed in the two studies. While Handel and Oshinsky combined responses where participants tapped every other element of a pulse train with the corresponding pulse train responses, such “skipping-a-beat” responses were assigned a separate category in the present study. Another cause of the discrepancies could be differences in the stimuli. In the stimuli used by Handel and Oshinsky, the duration of individual elements in the pulse trains was always equal to half the duration of the IOIs, such that pulse trains with fewer elements consisted of longer sounds. In comparison, all pulse trains in the present study were composed of the same short cowbell sound to avoid stimulus duration confounds. It is possible that the resulting binary structure of all pulse train elements in the stimuli used by Handel and Oshinsky has weakened the propensity towards binary subdivision of the beat.”

- "tapping preference transition points were tightly linked to the tempo of the subdivisions, rather than to the tempo of the pulse trains" - this is a really neat and important point from the Tempo experiment, but it is not actually at all obvious from fig 3-4. I wonder if you could somehow show this in a figure. perhaps the "stacked" design of the stimuli (Table 1) where an interval that is the "3" in one stimulus is the "2" in another can be exploited to make this point?

REPLY - Thanks for taking note of this. In the tempo experiment this point is most obvious at around 100ms / 600 BPM, i.e. the point at which elements at the subdivision level exceed the grouping rate limit. We have highlighted the relevant area of Figure 4 in the revised manuscript (see Figure 4 in our reply above). In the Ratio experiment we anchored the stimuli to the subdivision tempo, not the pulse train, and we did actually primarily have the Ratio experiment in mind when we wrote this sentence in the original manuscript. We acknowledge the need to clarify this point so we have added the following explanation to the Ratio Results and Discussion as well:

“A comparison of response distributions in the 2:3 and the 2:5 polyrhythms also reveals that tempo induced transition points, specifically the increases in *fast pulse train - half tempo* responses, are linked to the tempo of the subdivisions (shown on the x-axis of Figure 5C), not to the number of elements in the fast pulse train or to the overall cycle duration, both of which differs between the two polyrhythms.”

- general discussion paragraph 2: there may also be evidence for tick-tock effect in macaques: https://papers.ssrn.com/sol3/papers.cfm?abstract_id=3790895 also, it would be helpful to describe in more detail the specific findings in Savage et al 2015 regarding binary vs. ternary meters in music across cultures, and whether it is consistent with the findings here.

REPLY: The findings in macaques are indeed very promising, because they point to a similar perception of rhythm in other primates. Unfortunately, cross-species comparisons (though extremely interesting) fall beyond the scope of this manuscript. We narrow our discussion down to human beat perception and sensorimotor synchronization. However, in line with your suggestion, we added details about the specific findings reported in Savage et al. 2015:

“Preferences for binary groupings of beats appear in early stages of development (Bergeson & Trehub, 2006) and in adults (Drake & Bertrand, 2001), and simpler grouping of beats are statistical universals across musical cultures (Savage et al., 2015). A binary categorization of events emerges even spontaneously when irregular rhythms are imitated from participant to participant in iterated learning paradigms (Ravignani et al., 2016), likely due to an automatic subdivision of the beat into two intervals (Repp, 2008). Indeed, sensorimotor synchronization to a periodic beat is generally more consistent when it is subdivided into two events, compared to three, four or five events; an advantage that can be observed in the brain when measuring the steady-state evoked potentials related to duplets (Celma-Miralles et al., 2021). **Note, that direct comparisons between studies can be difficult because the grouping processes described may pertain to different metrical levels (subdivision, beat, or cycle level). Savage and colleagues (2015) listed the presence of “2- or 3-beat subdivisions” as the first statistical universal in a sample of 304 cross-cultural music recordings. While this description could be interpreted as referring to the subdivision level, closer inspection reveals that it does in fact mean subdividing the cycle into two or three beats. Consequently, this statistical universal holds no information as to whether these beats admit binary or ternary subdivision. Nonetheless, the propensity towards simplicity shown in all of the aforementioned studies align well with our interpretation that beat perception follows a subdivision hierarchy in favor of binary groupings, the simplest organization of temporal information.**”

- though this is a behavioral study, it is odd that there is no discussion at all on possible neurophysiological accounts for the preference for binary subdivision. could differences in beat salience of the different groupings account for the data (Rajendran, et al 2017)? Are the data consistent with tapping+EEG studies of beat perception (Nozaradan et al 2011;2012)? Would neural resonance theory predict a preference for binary groupings (Large et al 2015)?

REPLY – thanks for yet another relevant point which was left out of the original manuscript in an attempt to narrow down the discussion. We are happy for this opportunity to touch upon it nonetheless. We added the following section to the addition to the General Discussion regarding cross-cultural work.

“Claims of universality of a perceptual phenomenon can be supported by neurophysiological evidence. The current study did not collect neurophysiological data and cannot directly discuss the universality of the results. Yet, recent evidence for the presence of oscillatory dynamics in auditory cortex during rhythm processing align well with our findings of a preference for simple subdivision groupings (Doelling et al., 2019). Neural resonance models that describe the brain as a system of coupled non-linear oscillators predict more power at frequencies closer to the stimulus frequency, i.e., in our context more power at frequencies related to binary compared to ternary subdivisions (Large, 2008). Such higher-order resonance could explain why the peak was larger at the frequency related to the 3-beat (binary) than the 2-beat (ternary) in the 2:3 polyrhythm presented to participants in a study by Chemin and colleagues (2014). Overall, higher-order resonance phenomena in the brain provide neurophysiological explanations for the preference for 1:2 over 1:3 temporal relationships (Large, 2008), which in turn may explain why polyrhythms are not perceptually ambiguous.”

Typos:

under "Participants", 4th line: "youth countries"? also, the percentages don't add up

REPLY: Thank you for spotting this bug. It was not 62.3% but 100-62.3= 37.7%. We changed the sentence to:

“The remaining 37.7 % of participants grew up in forty-four different countries.”

under "Statistical analyses", 2nd line: fast pulse train is repeated

REPLY: We could not find this typo?
